# Malignant gastric outlet obstruction: direct biopsy in the submucosal tunnel to obtain the diagnosis

**DOI:** 10.1055/a-2307-5973

**Published:** 2024-07-03

**Authors:** Liansong Ye, Nuoya Zhou, Yi Mou, Chao Zhang, Lihong Wei, Xinhua Zhang, Bing Hu

**Affiliations:** 1 34753 Gastroenterology and Hepatology, Digestive Endoscopy Medical Engineering Research Laboratory, West China Hospital, Chengdu, China; 2 Gastroenterology, Xi-an No. 3 Hospital affiliated to Northwest University, Xiʼan, China; 3 Traditional Chinese Medicine, Hangcheng Peopleʼs Hospital, Weinan, China; 4 608062 Gastroenterology, The First Peopleʼs Hospital of Xianyang, Xianyang, China

A 59-year-old man presented with recurring early satiety for 1 year and postprandial vomiting for 2 months; he had also experienced weight loss of approximately 12 kg over the previous year. Gastroscopy showed food retention in the gastric cavity and an endoscope with a diameter of 8.9 mm could not be passed through the pylorus ( Fig. 1Fig. 1 ). The mucosa of the pylorus appeared normal. A barium swallow showed delayed emptying of the stomach, with no filling defects or niches ( Fig. 2Fig. 2 ). Abdominal computed tomography showed localized thickening of the gastric antrum, without enlargement of the lymph nodes ( Fig. 3Fig. 3 ). Endoscopic ultrasonography (EUS) showed thickened muscularis propria at the pylorus ( Fig. 4Fig. 4 ). Given the presumed diagnosis of hypertrophic pyloric stenosis, and after the patient had given informed consent, we performed peroral endoscopic myotomy (POEM) ( Video 1Video 1 ).

**Fig. 1:**
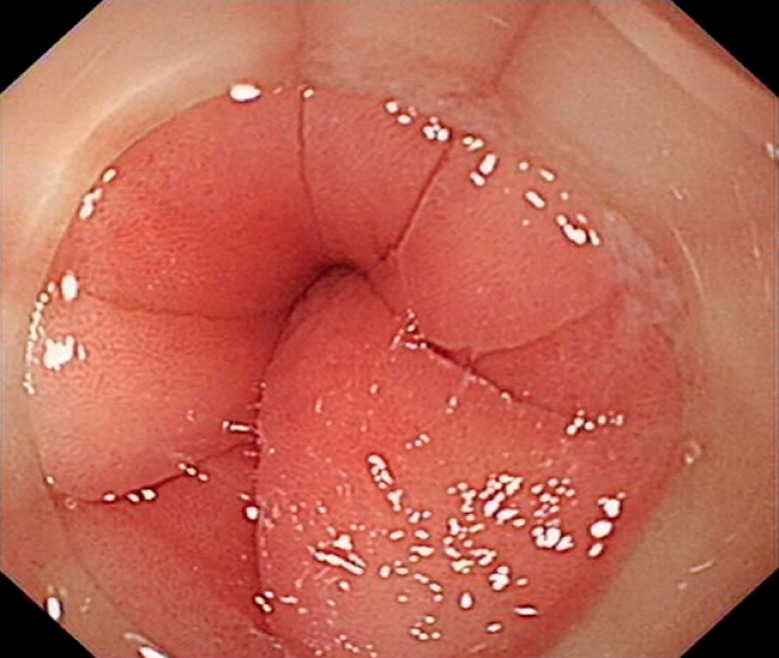
**Fig. 1** Endoscopic image showing the normal-appearing mucosa of the pylorus, although the endoscope could not be passed through to the duodenum.

**Fig. 2:**
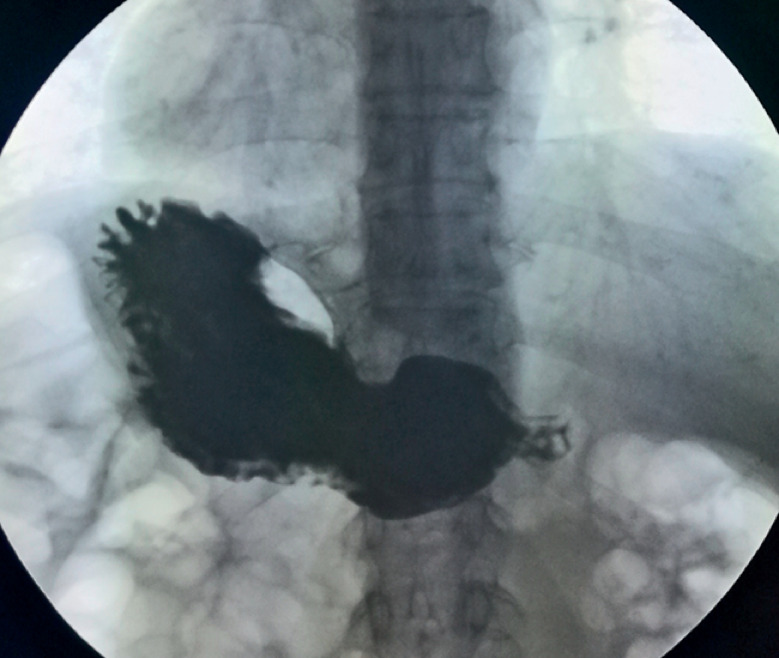
**Fig. 2** Radiographic image from a barium swallow showing delayed emptying of the stomach, with no filling defects or niches.

**Fig. 3:**
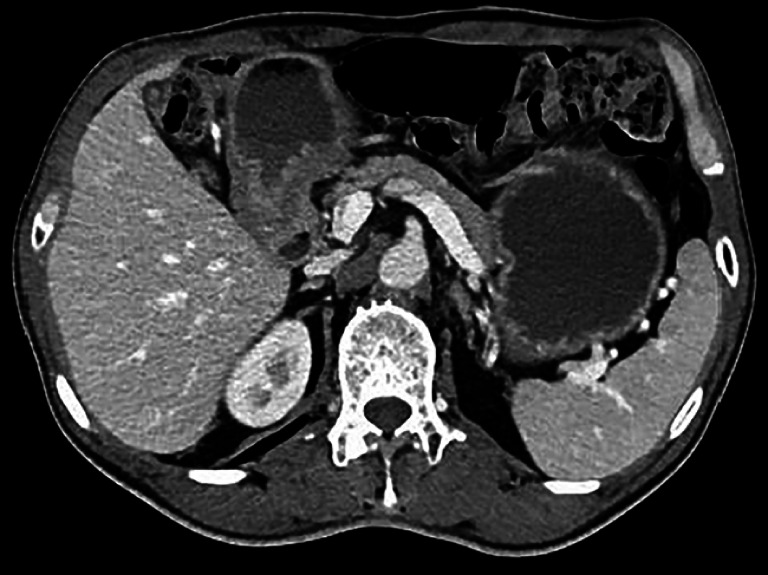
**Fig. 3** Abdominal computed tomography image showing localized thickening of the gastric antrum, with no enlarged lymph nodes.

**Fig. 4:**
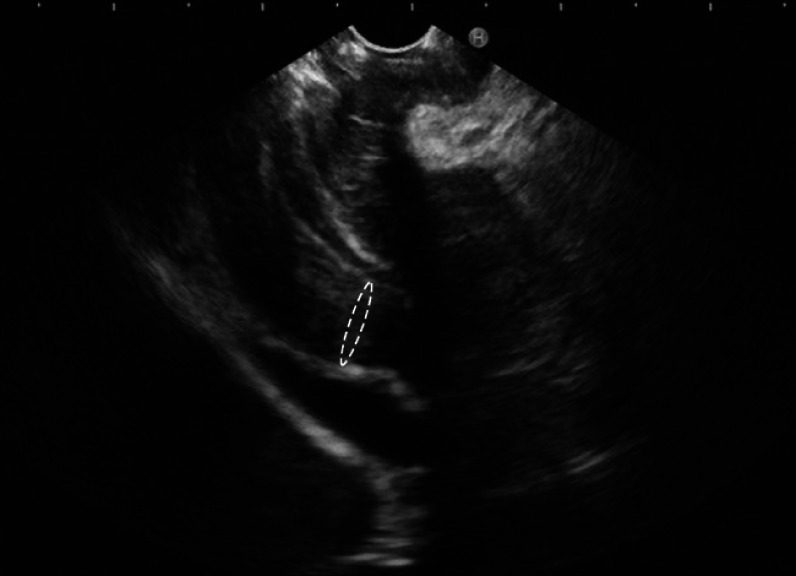
**Fig. 4** Endoscopic ultrasonography image showing thickening of the muscularis propria at the pylorus.

A direct biopsy is performed in the submucosal tunnel to obtain diagnostic tissue in a case of malignant gastric outlet obstruction.Video 1Video 1

Submucosal injection was performed on the posterior wall 8 cm proximal to the pylorus. A submucosal tunnel was subsequently created, but the procedure was interrupted because of dense adhesions of the thickened whitish muscularis propria and superficial mucosa. A sample of tissue from the thickened muscularis was obtained for pathology using a snare. Pathological findings subsequently showed the presence of atypical cells ( Fig. 5Fig. 5 ), and immunohistochemistry demonstrated that these atypical cells were positive for PCK and CK8, confirming a poorly differentiated gastric adenocarcinoma. The patient underwent surgical intervention, followed by systemic chemotherapy, but tumor recurrence was detected within 1 year.

**Fig. 5:**
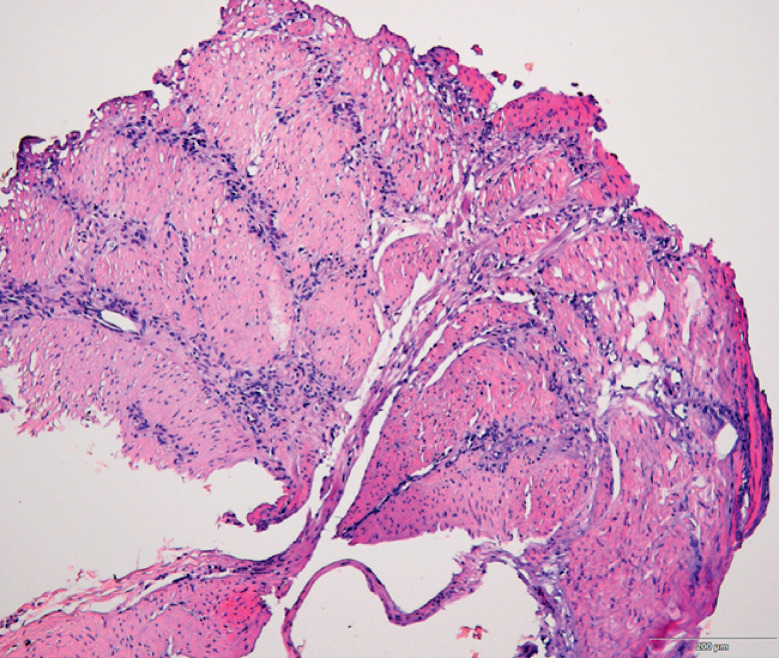
**Fig. 5** Histological appearance of the biopsy of the thickened muscularis propria showing several atypical cells in the muscular tissue.

Gastric outlet obstruction caused by gastric carcinoma is common in clinical practice 11 ; however, in this case, the advanced gastric carcinoma did not present with one of the commonly seen growth patterns, such as a polypoid, fungating, ulcerating, or diffusely infiltrating lesion 22 , and the superficial mucosa above it was normal, which made the preoperative diagnosis difficult. Techniques such as the taking of deep samples via ESD that allow the full submucosa to be sampled could help with diagnosis. As per our experience, direct biopsy in the submucosal tunnel can also help make the final diagnosis.

Endoscopy_UCTN_Code_TTT_1AO_2AC

## Notes

Endoscopy E-Videos https://eref.thieme.de/e-videos
*E-Videos* is an open access online section of the journal *Endoscopy* , reporting on interesting cases and new techniques in gastroenterological endoscopy. All papers include a high-quality video and are published with a Creative Commons CC-BY license. Endoscopy E-Videos qualify for HINARI discounts and waivers and eligibility is automatically checked during the submission process. We grant 100% waivers to articles whose corresponding authors are based in Group A countries and 50% waivers to those who are based in Group B countries as classified by Research4Life (see: https://www.research4life.org/access/eligibility/ ). This section has its own submission website at https://mc.manuscriptcentral.com/e-videos .

## References

[1] Fukami N Anderson MA Khan K The role of endoscopy in gastroduodenal obstruction and gastroparesis Gastrointest Endosc 2011 74 13 21 10.1016/j.gie.2010.12.003 21704805

[2] Hu B El Hajj N Sittler S Gastric cancer: Classification, histology and application of molecular pathology J Gastrointest Oncol 2012 3 251 261 10.3978/j.issn.2078-6891.2012.021 22943016 PMC3418539

